# Safety of Food and Water Supplies in the Landscape of Changing Climate

**DOI:** 10.3390/microorganisms7100469

**Published:** 2019-10-18

**Authors:** Aliyar Cyrus Fouladkhah, Brian Thompson, Janey Smith Camp

**Affiliations:** 1 Public Health Microbiology Laboratory, Tennessee State University, Nashville, TN 37209, USA; 2 School of Public Health, Yale University, 60 College St, New Haven, CT 06510, USA; brian.thompson@yale.edu; 3 Department of Civil and Environmental Engineering, Vanderbilt University, Nashville, TN 37235, USA; janey.camp@vanderbilt.edu

In response to evolving environmental, production, and processing conditions, microbial communities have tremendous abilities to move toward increased diversity and fitness by various pathways such as vertical and horizontal gene transfer mechanisms, biofilm formation, and quorum sensing [1,2]. As such, assuring the safety of water and food supplies from various natural and anthropogenic microbial pathogens is a daunting task and a moving target. Recent outbreaks of *Listeria monocytogenes* in South Africa associated with a ready-to-eat product (affecting close to 1000 individuals) and the 2018 outbreak of Shiga toxin-producing *Escherichia coli* O26 associated with ground meat in the United States (leading to the recall of more than 132,000 pounds of products) are bitter reminders of the devastating influences of foodborne diseases on the public health and food manufacturing [3,4].

Recent epidemiological studies of world populations indicate that 420,000 people lose their lives every year due to foodborne diseases, with around one-third of those being 5 years of age or younger. It is further estimated that every year, 1 in 10 individuals experience foodborne diseases around the globe, leading to an annual loss of 33 million healthy life years [5]. These episodes of food and water illnesses, hospitalizations, and deaths are concerns for both developing economies and developed nations. In the United States, as an example, epidemiological data derived from active surveillance data of the Centers for Disease Control and Prevention reveals that every year 31 main foodborne pathogens cause 9.4 million episodes of illnesses and about 56,000 cases of hospitalizations, leading to at least 1351 deaths of American adults and children [6].

In addition to these public health challenges, foodborne diseases are a major cause of consumer insecurity and economic burden to private industry, healthcare facilities, and government agencies due to costs associated with medical treatments and secondary costs related to food recalls and outbreak investigations [1]. Foodborne nontyphoidal *Salmonella enterica* serovars, as an example, cause an estimated 1,027,561 illnesses annually in the United States, with 27.2% and 0.5% hospitalization and death rates, respectively [6], leading to annual public health burden of 32,900 disability-adjusted life years [7]. Similarly, from 1998 to 2018, the bacterium had been the causal agent of >2500 single or multi-state outbreaks in the United States [8]. Overall, the cost of foodborne diseases is estimated to be $77.7 billion annually in the United States [9].

In addition to economic losses, consumers’ insecurity, and hospitalization, illness, and death episodes, victims of foodborne diseases may suffer prolonged and potentially life-long health complications after exposures to foodborne pathogens. Some of these main sequelae are Guillain–Barré syndrome, reactive arthritis, post-infectious irritable bowel syndrome, hemolytic uremic syndrome, and end-stage renal disease that could occur after infections with foodborne pathogens such as *Campylobacter* spp., *Salmonella enterica* serovars, and various serogroups of Shiga toxin-producing *Escherichia coli*. These additional public health burdens are calculated using epidemiological metrics such as the above-mentioned disability-adjusted life year [7].

Changes in the climate will unequivocally have pronounced effects on the proliferation of microbial pathogens and consequently the prevalence of foodborne diseases. As an example, it has been reported that only a 1 ºC increase (above 5 ºC) in temperature of an environment could lead to 5% to 10% increase in cases of salmonellosis [10]. In the United States alone, a 5% increase in illness episodes could translate to >50,000 additional cases of illnesses of nontyphoidal *Salmonella* serovars every year.

Similarly, the safety of water supplies is also interconnected with the changing climate. The World Health Organization estimates that approximately 2 million deaths each year are attributed to waterborne diarrheal diseases, with the vast majority of these deaths occurring in children [11]. This is largely attributed to the fact that 785 million people lack basic drinking-water service, with 144 million of these people reliant upon surface water [12]. Climatic conditions such as flooding and drought can influence the fate and transport of pathogenic microorganisms, as well as their fate and proliferation rates in the environment. The potential impacts of climate change on water supplies are primarily centered on anticipated changes in precipitation and increasing temperatures.

Increased precipitation can lead to runoff and flooding. Increased nutrient loading of surface waters due to runoff in both urban and rural areas coupled with warm temperatures can contribute to increased multiplication of cyanobacterial blooms and their harmful counterparts [13,14,15]. Flooding is attributed to increased risk of gastrointestinal illness when ground and surface sources for drinking water are impacted and not treated sufficiently. This presents potential concerns for citizens worldwide that do not have access to treated drinking water and may also present challenges in conventional treatment processes. During flooding events, surface and ground waters can become contaminated by sewer flooding and overflows that can result in higher risk of exposure to enteric pathogens [16]. In fact, there is a significant historic correlation between extreme rainfall events and outbreaks of waterborne diseases [17]. Conversely, drought can affect river flows, flushing rates, and eutrophication processes, which can lead to increased concentrations of Cyanobacteria and pathogens attributed to diarrheal diseases [11,18].

Surface water temperatures in streams have been shown to directly correlate to ambient air temperatures [19,20]. Therefore, one can anticipate that increasing ambient temperatures caused by climate change will in turn increase temperatures of surface waters, which serve as sources for drinking water, agricultural irrigation, and other domestic purposes that impact human health, especially in developing nations where drinking water treatment might not be as ubiquitous.

Climate change is one of the most significant challenges facing the public health and the safety and security of our food and water supplies. Without a major overhaul of our current energy production, political, and transportation systems, there will continue to be massive greenhouse gasses (GHGs) emissions into the atmosphere, further driving the changes in the climate. Beyond that, inertia in the climate systems will force continued climate change irrespective of GHG emission abatements [21]. Therefore, it is imperative that we better understand the risks to the safety of our food and water supply posed by climate change for the conduct of vulnerability assessments and the development of climate mitigation, adaption, and resilience programs.

Human-emitted GHGs are driving climate change [22] and altering many planetary systems in potentially irrevocable ways (e.g., the melting of the cryosphere, the warming of the oceans, changing rainfall patterns, etc.) [23]. Given that the climate will continue to warm throughout at least the first half of the 21st century [21], it is crucial to project the effects of future climate change. The Intergovernmental Panel on Climate Change (IPCC) has projections on the climatic effects of climate change across a range of different GHGs emissions scenarios (i.e., RCP2.6, RCP4.5, RCP6.0, RCP8.5) [23]. More GHGs emissions will result in an increased average surface temperature, greater precipitation, and higher sea levels. These consequences of future climate change can work individually or synergistically to threaten the safety of our food and water supply by impacting the fate and proliferation of foodborne and waterborne pathogens.

While the direct link between climate change and infectious diseases is inherently not characterized [24,25], we can infer their relationship by assimilating the impact of climatic factors and these diseases [26]. Many foodborne and waterborne diseases show strong cyclical periodicity based on precipitation and temperature—factors that are impacted by climate change [26,27]. The large rainfall events that will become commonplace due to climate change will challenge the safety of our water supply by causing sanitary and combined sewer overflows [28,29,30]. Further, these large rainstorms spread etiological agents of viral, parasitic, and bacterial infections [20,26,31]. To highlight one challenge, climate change is increasing sea surface temperature and causing sea level rise, both of which could fuel cholera outbreaks [32]. The increases in sea surface temperature promote greater *Vibrio* multiplication, as an example, and the rises in sea level could facilitate *Vibrio* infiltration into local water sources.

The public health and our food production and processing infrastructures in the 21st century will undoubtedly face paramount challenges due to global warming and subsequent changes in environmental conditions. Emerging and re-emerging zoonotic infectious diseases and subsequently increases in pesticides and veterinary drugs use and residues; increases in the prevalence of drug-resistant microorganisms in the food chain and healthcare facilities; the enhanced proliferation and prevalence of waterborne and foodborne bacteria, viruses, and parasitic agents in various regions and commodities; increases in the prevalence of toxigenic fungi and mycotoxins in the production environment and the food and feed chain; and increases in harmful algal blooms affecting fishery products will undoubtedly represent crucial challenges to our water and food safety and security in the 21st century. These will almost certainly affect the vulnerable populations from developing countries the most—those who have contributed the least to the current changes in the climate. Susceptible and at-risk populations, including the very young, elderly, pregnant women, and the immunocompromised, will also be most severely affected by this main public health challenge of our time.

Without intervention at the population level, the availability, access, utilization, and stability of an array of food and agricultural crops and water resources could almost certainly be jeopardized in the landscape of changing climate [33]. Although solutions to these challenges are inherently a moving target, the genetic wealth of plant, animal, and aquatic species could be a great resource for the development of climate resilience, adaption, and mitigation programs [34]. Developing evidence-based food and agricultural systems for climate change mitigation, expanding adaption programs tailored for small and emerging entrepreneurs, strengthening regional and international cooperation, and financing climate-smart food and agricultural systems are some of the current proposed interventions [35].

The current special issue provides a collection of research and review articles that discuss mitigating and prevention strategies associated with some of the most important foodborne and waterborne pathogens in the United States and around the globe. The public health burden of these pathogens will continue to gain further importance and momentum in future years in the landscape of the changing climate.
